# Vital Statistics of the Sandwich Islands

**Published:** 1878-06

**Authors:** John Dean Caton

**Affiliations:** Honolulu, Hawaiian Islands


### Correspondence.

---

Honolulu, Hawaiian Islands, February 16, 1878.

*To the Chicago Medical Journal and Examiner:*

Among the interesting observations I have been enabled to make in these Islands, I have gathered such facts as were within my reach, of vital statistics, with some extraordinary results, which may prove of interest. My attention was first attracted to the subject, by listening to a septennial sermon preached by Rev. Mr. Freer, pastor of the Congregational church in Honolulu, on the last Sunday of 1877. He stated that during the seven years in which he had had the charge of that church, there had been born to its members eighty children, and that of those under his charge, five under fifteen years of age had died of disease and one from accident, and that during the same time fourteen adult members of the church had died.

These statistics were not sufficiently full to satisfy me, so I took an early opportunity to call upon the reverend gentleman and told him that his discourse had awakened an interest in me which it did not satisfy, and that he would lay me under great obligations if he could inform me what was the average number, during the seven years, of the children under fifteen years of age of whom but five had died of disease, and what was the average number of adult members of his church, of whom fourteen had died in the same period, and also how many of the eighty children born during that time were still living. He expressed great willingness to do this as soon as he could make the necessary investigation, and when practicable he obligingly informed me that the average number, for the seven years, of the children from whom the five were taken, was 300. That the average number of the adult members of the church during the seven years, of whom the fourteen had died, was two hundred. That three of the five children who had died were of the eighty born during that time, leaving seventy-seven of them still living. He then added that no death had occurred during his time, among either class, of a person between the ages of six and eighteen years, so that the number of juveniles might have been largely increased by including all in that class under eighteen years of age. I remarked that I thought his statements most extraordinary, as I had seen it stated by high authority that one-half of the children born in the United States and in Europe die before they attain the age of five years. I asked him if he had ever applied figures to his facts so as to enable him to comprehend the results more clearly. He said he never had, for it had never occurred to him to do so. I then said, let us assume that the three hundred children represented 300 years of life each year, then during the seven years there were represented twenty-one hundred years of juvenile life, and if we divide this number by five, the number of deaths from disease, which has occured among them during that time, then there has less than one death occurred for four hundred years of juvenile life in his congregation, and by applying the same mode of computation to the adults of his church and the fatality among them during the same time, then there had occured one death for each one hundred years of adult life.

These results seemed very extraordinary he admitted, but that could not change the facts. At the first opportunity I laid these statements before Dr. McGrew, a physician of very extensive practice in Honolulu, who said that he did not doubt the truth of the statements, and that he did not think the results exceptional among the white population in the Islands. That the congregation of the Fort Street Church consisted of white people with very few exceptions, and that the few natives who worshiped in that church were of an exceptional class. That while the death of a white child in the Islands was of very rare occurrence the very reverse was the case among the native population. I asked the doctor how he explained the remarkable difference in the health of the children in the two races. He ascribed the difference to two principal causes: first the lack of constitutional vigor or vitality in the native children, or inherited weakness or disease, and second a lack of that care which the white children receive, or in other words a lack of maternal affection. He stated that all children here are subject to those juvenile diseases which afflict children in other countries, such as the whooping-cough, measles, and the like, but that here they are of a much milder type than in any other country of which he had any knowledge, and when proper care was taken of the patients, they very rarely proved fatal. That indeed the death of a white child was of very rare occurrence, so that parents become at least partially divested of that deep solicitude or rather apprehension which is felt in other countries, that the new born infant may not live to muturity. I then inquired if he could discover that this imumnity seemed to have a tendency to beget in the white mothers, an inclination to neglect their children. He very promptly answered, not in the least; that the white mothers here are as devoted to their offspring as in any country in the world, only that they were more richly rewarded here for such affectionate care than elsewhere.

While upon the Islands I have neglected no opportunity to push my inquiries on this interesting subject, and found the results nowhere less favorable than the first statements given. In a conversation with Dr. Endor, formerly of St. Louis, now a practicing physician at Wailuku on the Island of Moni, I learned that by applying the same mode of computation first adopted to his facts, there were six hundred years of juvenile life to each death of a child among the white population within his practice, while his observations corresponded with those of Dr. McGrew as to the native population.

Rev. S. C. Damon, seamen's chaplin at Honolulu for these many years, whose hand seems to be in every good work, took me to visit the Punahou school and Oahu college which is situated just beyond the city. From a historical essay, read at a meeting of the alumni of this institution by Hon. A. F. Judd, in 1866, I learn that "the school opened on the 11th of July, 1841, with an attendance of thirty-four pupils solely the children of missionaries." "The greatest number (of pupils) in any one year was in 1858, when there were 77. In 1861 there were 76 and in the year just closed (1865), 51. Total number of pupils during twenty-five years, 290, number of deaths so far as known, 20, leaving alumni alive 270." I was informed that a large percentage of these deaths occurred out of the Islands. At the time of my visit the school had been in operation over 36 years, and not a single death had ever occurred among the pupils. I could not obtain the exact average attendance but Dr. Damon expressed the opinion that the average would be about 70. While the data obtained lack precision in some important particulars, as for instance, the number of deaths of the alumni which occurred on the Islands, and the average number of pupils in the school, still they are valuable as tending to show the healthfulness of the Islands.

I have made some efforts to get a biological history of all the missionary families in these islands, but without success. This, complete, would form a most interesting chapter in Natural History, and such a work I believe not impracticable at this day. It would show a measure of generation and health, and periods of vitality, rarely to be paralleled in the history of the human family. I have a publication which advances a step in this direction. The missionary mothers in the islands formed themselves into a society which they called the "Maternal Association of the Sandwich Islands;" and in 1854 published a list of the members and children of the association called the *Blue Book.* In this the dates of the births of the children and the dates and the places of the deaths of members and children are given. In this book are given sixty-five missionary mothers and their children, of whom there were born 288. Thirty-two of these had died in the Islands of disease, one was drowned, and five had died at other places, one of whom was drowned. Of the 65 matrons, nine had died in the Islands, and two at other places. The first child was born in October, 1820; and the last in March, 1854. The value of these figures is better appreciated when we remember that the first missionaries arrived here in 1818, so that the period embraced in the statistics was 36 years. I regret that I have not the means of determining the numbers and dates of the arrivals of the reinforcements, but as the dates of the births of the children are given, together with the dates and places of the deaths among them, we are enabled from these to make valuable computations. As we have seen, the 65 mothers bore 288 children, of whom 38 had died. Reckoning from the birth of each child up to 1854, the time when the statement was made, the aggregate years of life, if none had died, was 4100; computing then the time from each death up to 1854, the aggregate is found to be 463 years, which, if deducted from the 4100 years, leaves 3637 years of life. With these data given, each one may make his own computation, though I cannot help observing that more than 86 per cent. of those children born in the Islands, were living at the end of the period of 34 years after the first was born. The medical profession may be familiar with such results but I confess they surprised me.

I may state here that I learned from accoucheurs and other reliable sources that parturition is much easier and safer here than is usual in other countries, and that even a few months residence on the Islands seemed to prepare the system for such results.

While enjoying the hospitality of Father Alexander at Wailuku, and before I had seen the *Blue Book*, I expressed a desire to obtain a biological history of the missionary families of the Islands, when he remarked that there might be some difficulty in getting the history of all, but he could readily give the history of one. That he and his wife came from Kentucky to the Islands as missionaries in 1832. That they had had nine children, nineteen grandchildren and six sons and daughters-in-law, and that there had never been a death in the family, but all were now living on the Islands, "and," said he, as he straightened himself up, a just pride beaming from every lineament of his countenance, "not a black sheep among them all." The venerable heads of that remarkable family still manifest the elasticity and vigor of middle life, and labor with unabated zeal in the cause to which they at the beginning consecrated their lives. Truly they have been conspicuously blessed.

The departments of the government kindly furnished me with all the statistical information in their offices, but I find nothing which answers the inquiries for vital statistics which I am making. The rapid diminution of the native population, which has long been known the world over, and has been often the subject of elaborate dissertations, had not led me to expect here the most favorable conditions for human life to be found in any country of which I have any knowledge. It is not only remarkable as a sanitarium for children, but for adults as well. The aged missionaries who have been laboring here for forty or fifty years or more, I find still strong and vigorous, working zealously in their missions, with a promise still of many years of usefulness, notwithstanding the hardships and privations through which they have passed.

Let me add that the last census shows, for the first time, an increase of births over deaths of the native population in Honolulu, but I fear the hopes which this statement inspired, that a turning point had been reached in the vitality of the native population of these Islands, will prove illusory, and that they are at last destined to fade away and disappear.

Yours, JOHN DEAN CATON.

